# PHO1: linking phosphate nutrition translocation and floral signalling in plants

**DOI:** 10.1093/jxb/erae302

**Published:** 2024-08-22

**Authors:** Cunman He, Huixia Shou

**Affiliations:** The Provincial International Science and Technology Cooperation Base on Engineering Biology, International Campus of Zhejiang University, Haining, Zhejiang 314400, China; The Provincial International Science and Technology Cooperation Base on Engineering Biology, International Campus of Zhejiang University, Haining, Zhejiang 314400, China

**Keywords:** Flowering, higher plants, PHO1, phosphate, translocation

## Abstract

This article comments on:

**Dai S, Chen H, Shi Y, Xiao X, Xu L, Qin C, Zhu Y, Yi K, Lei M, Zeng H.** 2024. PHOSPHATE1-mediated phosphate translocation from roots to shoots regulates floral transition in plants. Journal of Experimental Botany **75**, 5054–5075. https://doi.org/10.1093/jxb/erae222

**Arabidopsis *PHOSPHATE1 (pho1*) is the first mutant isolated that affects inorganic phosphate (Pi) homeostasis (** **Poirier *et al*., 1991** **). PHO1 is predominantly expressed in the root stele and transfers Pi from roots to shoots (** **Hamburger *et al*., 2002**; **Rouached *et al*., 2011). In this issue, Dai *et al*. (2024) describe that PHO1 regulates flowering time by repressing the expression of *FLOWERING LOCUS T* (*FT*) and *TWIN SISTER of FT* (*TSF*) in a jasmonic acid (JA)-dependent manner. The results not only prove that PHO1 links Pi nutrition with flowering time, but also suggest that plant flowering time could be regulated by the supply of phosphorus.**

Phosphorus is an essential macronutrient required for plant growth and development. A phosphate-responsive signalling pathway, known as the PHO pathway, has been extensively studied and many components were identified (Fig. 1). *PHO1*, encoding a SYG1/PHO81/XPR1–ERD1/XPR1/SYG1 (SPX–EXS) protein, is well known to be involved in loading of Pi into the root xylem vessels, and transferring Pi from roots to shoots in Arabidopsis (Hamburger *et al.*, 2002). PHO1 protein abundance is adjusted via its degradation mediated by the ubiquitin-conjugating E2 enzyme, PHOSPHATE2 (PHO2) (Liu *et al*., 2012). The *PHO2* gene expression is post-transcriptionally repressed by phosphorus starvation-induced miRNA399 (miR399) with the induction of MYB transcription factors called PHOSPHATE STARVATION REGULATOR 1 (PHR1) and PHR1-like proteins (Bari *et al.*, 2006). SPX1 and SPX2 are direct Pi-dependent inhibitors of *PHR1* and negatively regulate its transcriptional activity in Arabidopsis (Puga *et al.*, 2014).

**Fig. 1.**
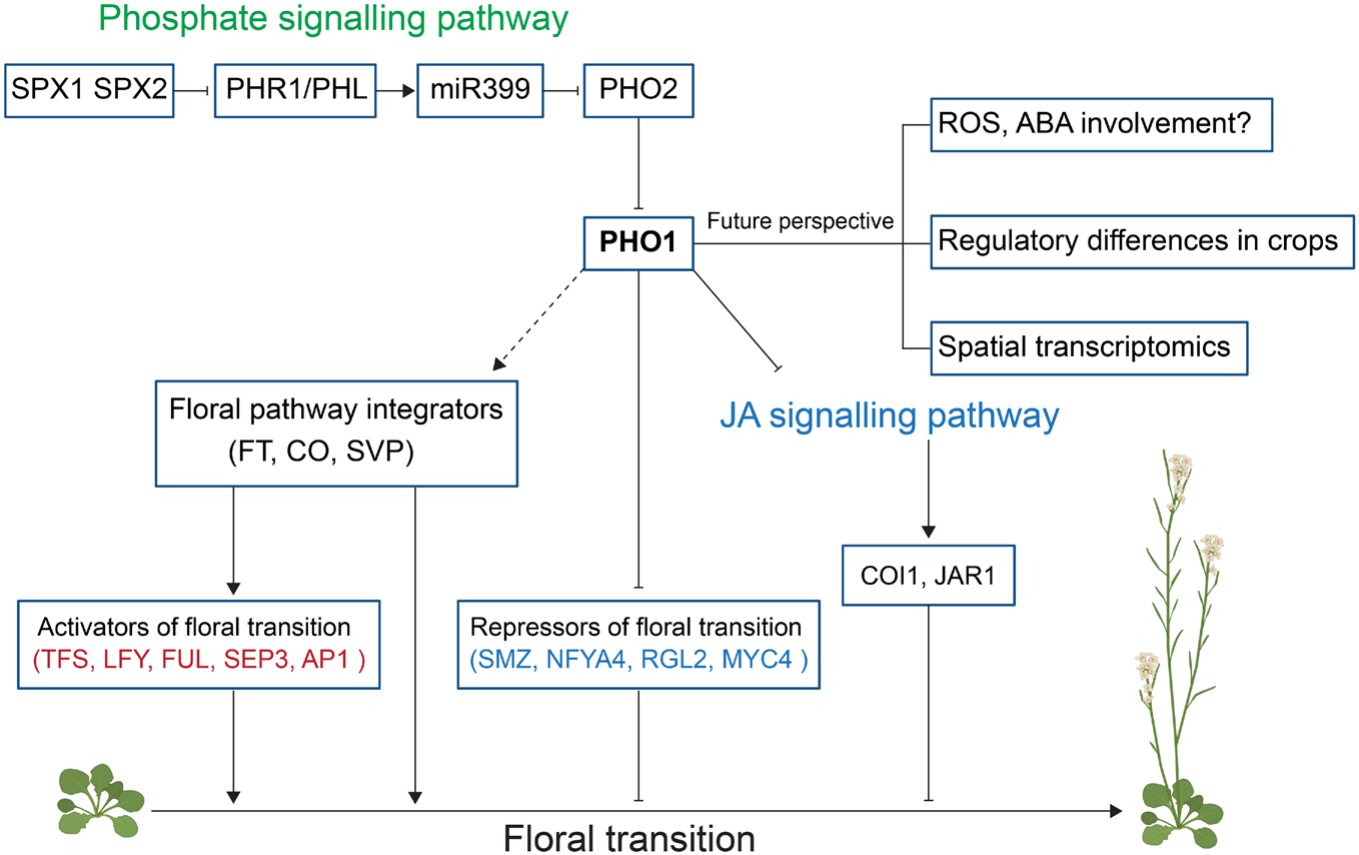
Defining and dissecting PHO1-mediated floral transition pathways in plants. The integration of the phosphate signalling pathway with floral transition by the action of PHOSPHATE 1 (PHO1), the jasmonic acid (JA) signalling pathway, and flowering pathway activators, repressors, as well as integrators. SYG1/PHO81/XPR1 (SPX)1 and SPX2 have a cellular Pi-dependent inhibitory effect on *PHR1*, and the expression of *PHO2* is post-transcriptionally repressed by miR399, and PHO2 regulates the stability of PHO1 by mediating its degradation. The expression of flowering pathway integrator *FLOWERING LOCUS T* (*FT*) is repressed in the *pho1* mutant and there is potential involvement of *CONSTANS* (*CO*) in PHO1-mediated flowering regulation under long-day conditions. Another flowering pathway integrator *SHORT VEGETATIVE PHASE* (*SVP*) may be required for the late flowering of *pho1* mutants. Five genes encoding activators of floral transition, namely *TARGET OF FLC AND SVP1* (*TFS1*), *LEAFY* (*LFY*), *FRUITFULL* (*FUL*), *SEPALLATA3* (*SEP3*), and *APETALA1* (*AP1*), were attenuated and four genes encoding repressors of floral transition, namely *SCHLAFMUTZE* (*SMZ*), *NUCLEAR TRANSCRIPTION FACTOR Y SUBUNIT A-4* (*NFYA4*), *RGA-LIKE 2* (*RGL2*), and *MYC4*, were induced in *pho1* shoots. The induction of the JA signalling pathway is responsible for the late flowering phenotype of *pho1* mutants with the involvement of *CORONATINE INSENSITIVE 1* (*COI1*)-dependent JA signalling pathway as well as *JAR1* (Dai *et al*., 2024). Future research could target three aspects: (i) whether other hormones and cellular processes could be involved in PHO1-mediated phosphate translocation regulating floral transition; (ii) the similarities and differences of PHO2, PHO1, and phosphate homeostasis-related components among different plant species and crops involved in floral transition; and (iii) using spatial transcriptomics to provide insights into how PHO1-mediated phosphate translocation regulates the floral transition in plants on a cellular level. Activators of floral transition are highlighted in red, and repressors of floral transition are indicated in blue. Arrows represent activation processes, and lines ending in bars indicate suppression processes. Paths illustrated with solid lines represent cases where experimental evidence exists for involvement; the dotted line indicates hypothetical involvement. The figure was generated with BioRender (https://biorender.com).

The regulation of flowering time in plants involves the integration of environmental stimuli and internal signals (Kinoshita and Richter, 2020). Various genetic pathways and molecular components contribute to this intricate regulatory network (Kinoshita and Richter, 2020). The central regulator in the photoperiodic pathway is CONSTANS (CO), a B-box zinc-finger transcription factor, which induces the expression of *FT*. FT, in turn, acts as a mobile floral stimulus, moving from leaves to the shoot apex to initiate flower formation (Shim *et al.*, 2017). The vernalization pathway involves the epigenetic down-regulation of *FLOWERING LOCUS C* (*FLC*) through exposure to prolonged cold temperature. FLC antagonizes the gibberellin (GA) pathway by repressing flowering promoters such as *FT* and *SUPPRESSOR OF CONSTANS OVEREXPRESSION 1* (*SOC1*) (Sharma *et al.*, 2020).

Nevertheless, the mechanism by which Pi nutrition is involved in floral transition regulation remains unclear. Using the *pho1* mutants, the study demonstrates that phosphorus nutrition in plants is a key factor influencing flowering (Dai *et al*., 2024).

Dai *et al.* (2024) found that two *pho1* mutants exhibited delayed flowering compared with the wild type (WT) under both long-day and short-day conditions. The late flowering phenotype of *pho1* mutants can be rescued by complementation with *pPHO1:PHO1-GFP*, which confirms that PHO1 plays a positive role in floral transition. By growing *pho1* and WT plants hydroponically with varying Pi concentrations, or spraying KH_2_PO_4_ solution onto the rosettes, the authors showed that the late flowering phenotype of *pho1* mutants is associated with low shoot Pi concentration. Pi supplementation, particularly at shoot apices, partially rescued this phenotype. Low Pi treatments repressed the flowering time of WT plants, emphasizing the significant role of Pi availability in regulating flowering time (Dai *et al*., 2024). Using a reciprocal micrografting experiment with WT and *pho1* mutant seedlings, the authors demonstrate that *PHO1* mutation in the roots fully explains the late flowering phenotype of *pho1* mutants. The molecular mechanisms underlying the modulation of flowering time by the upstream components of PHO1 were further investigated. The findings suggest a complex interplay of Pi signalling components in the regulation of flowering time (Dai *et al*., 2024).

An additional line of evidence for the link between Pi homeostasis and flowering time came from the identification of 15 genes associated with floral transition in the gene expression comparison of the *pho1* mutant with the WT under KCl, or KH_2_PO_4_ spray (Fig. 1). For instance, the expression of the well-known floral transition genes *FT* and *TSF* was dramatically reduced in *pho1* compared with the WT, which further confirmed the link between flowering time and phosphorus nutrition. The late flowering phenotype of the *pho1* mutant could be restored by overexpression of the *FT* gene.

## Is jasmonate signalling involved in the interaction of phosphorus nutrition and flowering time?

It is well known that jasmonic acid (JA) homeostasis and signalling play diverse roles in plant reproduction (Yuan and Zhang, 2015). The biosynthetic and signalling pathways of JA are relatively conserved among different plant species, but the role of JA in reproductive development is divergent (Yuan and Zhang, 2015; Han, 2017). In rice, JA promotes rice spikelet and stamen development. In contrast, JA signalling plays a negative role in floral transition in Arabidopsis (Zhao *et al.*, 2022).

Dai *et al.* (2024) found that many JA-responsive genes were differentially expressed in the *pho1* mutant when sprayed with KH_2_PO_4_ or KCl. This is consistent with the previous observation that Pi deficiency induces the JA pathway with involvement of PHO1 (Khan *et al.*, 2016). The late flowering phenotype of *pho1* mutants can be partially rescued by the mutation of *CORONATINE INSENSITIVE 1* (*COI1*), which is a key component of the JA perception machinery, and partially recovered by the knockout of JAR1 (JA-RESISTANCE 1) that is involved in JA biosynthesis. Thus, the induction of the JA signalling pathway is partially responsible for the late flowering phenotype of *pho1* mutants. Additionally, loss of function of *OsPHO1;2*, the rice homologue of *PHO1*, delays floral transition. The down-regulation of *Hd3a* and *RFT1* (the rice homologues of *FT*) in the *OsPHO1;2* mutant further underscores the conserved role of *PHO1* homologues in regulating flowering time across plant species (Dai *et al*., 2024).

## Future perspective

The plant hormone abscisic acid (ABA) and the balance of reactive oxygen species (ROS) in plant cells are associated with floral transition in plants (Wang *et al.*, 2013; Huang *et al.*, 2021). Gene Ontology analysis revealed enrichment of various biological processes and molecular functions among the differentially expressed genes in the *pho1* mutant compared with the WT, indicating that the impact of *PHO1* knockout might be not only on the JA pathway. The ABA-related genes *AIK1* (*ABA-insensitive protein kinase I*) and *AIRP2 (ABA-insensitive RING protein 2*), the auxin biosynthesis genes *YUC8* and *YUC9*, and the ROS homeostasis-related gene *ARS1* (*ABA & ROS sensitive 1*) were found to be differentially expressed in *pho1* compared with the WT (Dai *et al*., 2024). Thus, the loss of function of *PHO1* or changes in external Pi supply could influence ABA, auxin, and ROS responses, which may in turn affect the floral transition. Whether and how these hormones and cellular processes could rescue the late flowering phenotype of the *pho1* mutants remains to be determined in future studies.

Notably, Dai *et al*. (2024) found that knockout of *PHO2*, the upstream negative regulator of *PHO1*, led to early flowering under long-day conditions in Arabidopsis. However, rice *pho2* was shown to have delayed flowering phenotypes (Li *et al*., 2017). As the mechanism of post-translational degradation of PHO2 on PHO1 is common between rice and Arabidopsis (Liu *et al*., 2012), how did the difference in the effect of PHO2 on flowering in the two species arise? Is this related to the different effects of JA on flowering, or is the effect of PHO2 on regulation of the circadian clock and photoperiod-responsive genes between the long-day plant Arabidopsis and the short-day plant rice? Analysis of the expression of JA-responsive and flowering-related genes as well as involvement of the photoperiod pathway in both rice and Arabidopsis *pho2* mutants may solve the puzzle.

Single-cell and spatial transcriptomics are advanced techniques used in plant biology to understand gene expression at a highly detailed level (Rao *et al.*, 2021). Spatial transcriptomics can provide insights into how PHO1-mediated Pi translocation regulates floral transition in plants on a cellular level, how Pi-responsive genes are expressed in different tissues, how neighbouring cells interact in response to different Pi levels, and how the overall tissue architecture adapts to changes in Pi availability and regulates floral transition.

Overall, this study significantly contributes to our understanding of the function of PHO1 and phosphate translocation in floral transition and provides valuable insights into the molecular mechanisms governing flowering time regulation in plants. The proposed model for how PHO1-mediated phosphate translocation and signalling regulates floral transition in plants and future perspective is summarized in Fig. 1.
